# A Review of Attacks, Vulnerabilities, and Defenses in Industry 4.0 with New Challenges on Data Sovereignty Ahead

**DOI:** 10.3390/s21155189

**Published:** 2021-07-30

**Authors:** Vítor Pedreira, Daniel Barros, Pedro Pinto

**Affiliations:** 1 Instituto Politécnico de Viana do Castelo, 4900-347 Viana do Castelo, Portugal; vitorpedreira@ipvc.pt (V.P.); danielbarros@ipvc.pt (D.B.); 2 Universidade da Maia, 4475-690 Maia, Portugal; 3 INESC TEC, 4200-465 Porto, Portugal

**Keywords:** cybersecurity, attacks, defenses, industry 4.0, vulnerabilities, survey, data sovereignty

## Abstract

The concepts brought by Industry 4.0 have been explored and gradually applied.The cybersecurity impacts on the progress of Industry 4.0 implementations and their interactions with other technologies require constant surveillance, and it is important to forecast cybersecurity-related challenges and trends to prevent and mitigate these impacts. The contributions of this paper are as follows: (1) it presents the results of a systematic review of industry 4.0 regarding attacks, vulnerabilities and defense strategies, (2) it details and classifies the attacks, vulnerabilities and defenses mechanisms, and (3) it presents a discussion of recent challenges and trends regarding cybersecurity-related areas for Industry 4.0. From the systematic review, regarding the attacks, the results show that most attacks are carried out on the network layer, where dos-related and mitm attacks are the most prevalent ones. Regarding vulnerabilities, security flaws in services and source code, and incorrect validations in authentication procedures are highlighted. These are vulnerabilities that can be exploited by dos attacks and buffer overflows in industrial devices and networks. Regarding defense strategies, Blockchain is presented as one of the most relevant technologies under study in terms of defense mechanisms, thanks to its ability to be used in a variety of solutions, from Intrusion Detection Systems to the prevention of Distributed dos attacks, and most defense strategies are presented as an after-attack solution or prevention, in the sense that the defense mechanisms are only placed or thought, only after the harm has been done, and not as a mitigation strategy to prevent the cyberattack. Concerning challenges and trends, the review shows that digital sovereignty, cyber sovereignty, and data sovereignty are recent topics being explored by researchers within the Industry 4.0 scope, and GAIA-X and International Data Spaces are recent initiatives regarding data sovereignty. A discussion of trends is provided, and future challenges are pointed out.

## 1. Introduction

Industry 4.0, or the fourth industrial revolution, advocates the automation of traditional practices of manufacturing and industrialization [1], with the use of smart technologies allowing machine-to-machine communication [2]. This concept of industry assumes machine-to-machine and human-to-machine communications, using the latest methods, techniques and tools, intended to transform digitally the industries manufacturing, production, and value creation processes.

Recent technologies have been integrated within Industry 4.0 implementations, bringing new challenges in the cybersecurity area [3]. Recent Industry 4.0 implementations include technologies such as cloud computing, ai, cps or iot. iiot devices [4], iot devices intended for industrial use, are typically small-sized, low-cost, efficient, capable of having sensors or actuators [5], but featuring low computing power micro-controllers [6]. A cluster of these devices can control entire manufacturing processes with great efficiency, making them useful in large manufacturing companies.

With the increase of devices connected to Industry 4.0-enabled networks, the surface of attack also expands. Malicious actors may find in any smart device an open door to exploit new vulnerabilities and perform attacks on them or on their infrastructure, with the intent of impacting financially a company or industry [7]. When compromised, these devices can cause serious damage to material goods, such as products on a manufacturing line, or immaterial goods, such as the leakage of sensitive information or industrial secrets. Several attacks have targeted industrial facilities and their devices, from the Stuxnet [8] in 2010, to the Trojan BlackEnergy [9] in 2015 and Mirai in 2016 [10], to recent ransomware attacks, such as the WannaCry [11] in 2017 or the LockerGoga [12] in 2019, resulting in operational and financial impact for affected companies. Thus, it is relevant to constantly monitor cybersecurity risks, the impact of attacks and the state of defense mechanisms in Industry 4.0 implementations [13]. A round of efforts, such as the one described in [14], are focused on good practices and prevention to keep Industry 4.0 implementations and it systems secure, while ensuring their normal operation and maintenance.

The contributions of this paper can be divided into three. First, a general systematic review of current cybersecurity attacks, vulnerabilities and defenses in Industry 4.0 and 5.0 scenarios is presented. Second, a detailed analysis and categorization regarding attacks, vulnerabilities and defenses of selected studies is presented. Third, a discussion is presented of recent challenges and trends regarding these areas on Industry 4.0 and Cybersecurity. This review is divided into three steps: (1) General Review, (2) Abstracts Review, and (3) Selected Papers Review.

This systematic review allows the identification of the most common attacks, vulnerabilities, and defense strategies. Additionally, a set of challenges and trends regarding Industry 4.0 are highlighted as an effort to enhance the detection and prediction of new vulnerabilities or zero-day attacks, and creating the necessary defense mechanisms to protect industry data. Digital, Cyber and Data Sovereignty concepts are discussed since challenges emerge regarding data sharing and ownership, and recent initiatives such as ids [15] and GAIA-X [16] are promoting data exchange, with the objective of ensuring data security and sovereignty.

The remainder of this document is organized as follows: Section 2 introduces the methodology used for the systematic review; Section 3 presents the results for the General Review; Section 4 presents the results for the Abstracts Review; Section 5 reviews the selected studies; Section 6 draws a discussion relative to the results obtained; lastly, in Section 7 conclusions are made.

## 2. Review Methodology

This review around the cybersecurity-related topics in Industry 4.0 intends to overview types of attacks, vulnerabilities and defense strategies. Additionally, this study aims to identify whether topics such as data sovereignty, digital sovereignty and cyber sovereignty are current trends for cybersecurity in the context of Industry 4.0, due to the appearance of the ids and GAIA-X. Thus, a general systematic review was carried out, inspired by the methodology in [17], adapted for the current study context.

For the current study, we assume that the published paper progress over time (from 2014 to 2021) can be a possible approach to overview the cybersecurity-related topics and infer current trends and challenges. Thus, the following Research Questions (RQ) were formulated:

- RQ1—What is the progress of the cybersecurity area for Industry 4.0, in number of papers related to vulnerabilities, defense and attacks topics?
- RQ2—What is the progress, in number of papers for the intersections of the topics of vulnerabilities, attacks and defense mechanisms for Industry 4.0?
- RQ3—What is the progress of the number of papers for challenges and trends related to data sovereignty, digital sovereignty and cyber sovereignty areas for Industry 4.0?

After defining the research questions, search engines were chosen. For this study, we selected the ACM Digital Library, Scopus and IEEExplore databases to receive the queries as input and to provide quantitative results of the number of papers.

The keywords used to perform the search queries were defined to include the results of Industry 4.0 and Industry 5.0 and to gather all recent matches regarding attacks, vulnerabilities, defenses, and sovereignty-related terms. The primary and secondary keywords used are presented in Table 1. The keywords were used in search queries, where the primary keywords were searched for in the abstract of the paper and the secondary keywords were searched for in the all the metadata of the paper. The search queries used for the three databases can be found in [18].

**Figure 1:**
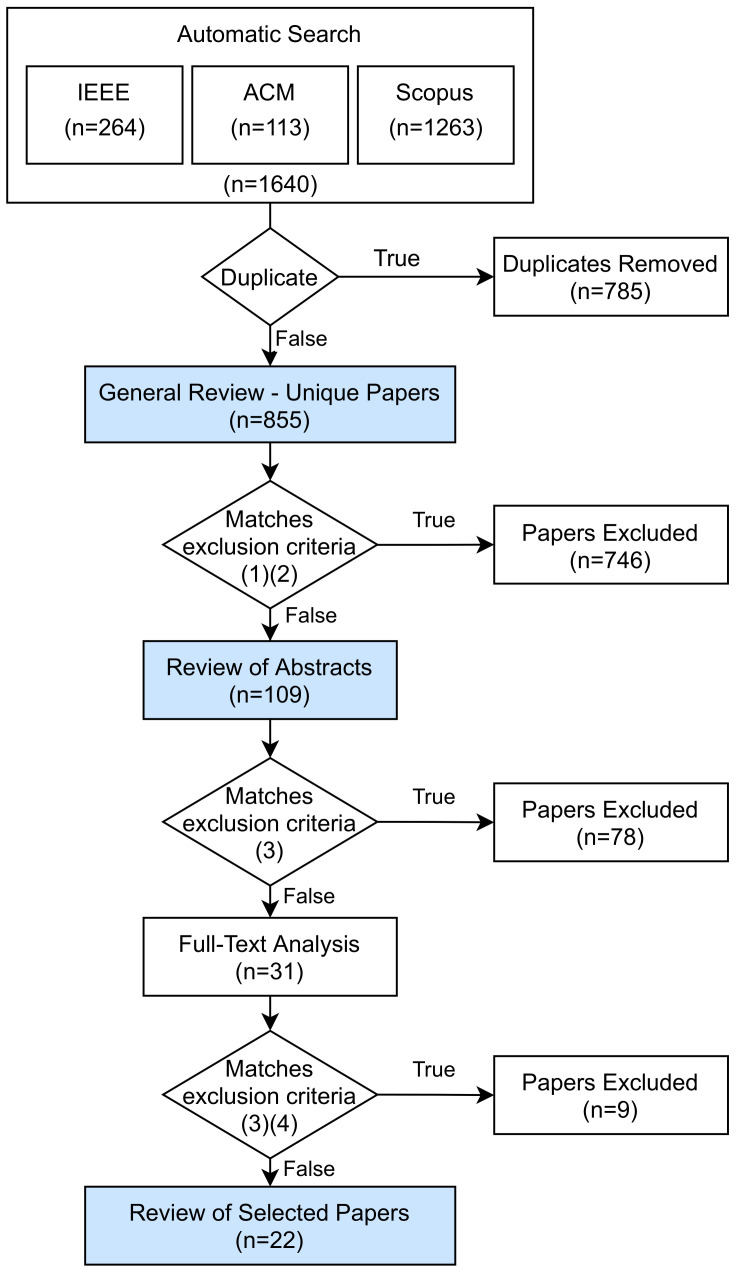
Systematic Review Methodology.

In Figure 1 the adopted systematic review process with the number of papers obtained in each stage is shown.

After the first search queries 1640 papers were selected, and duplicates were removed. For the remaining 855 papers, the StArt tool [19], a support tool for systematic reviews, was used. All 855 papers were imported and classified with a score assigned as follows: 20 points for each time one of the keywords appears in the title, 10 points for each time one of the keywords appears in the abstract and 5 points for each time one of the keywords appears in the full text. The application of the score resulted in a paper scores ranging from 0 to 260. Then, a set of exclusion criteria was used as presented in Table 2. The application of this criteria, which included the exclusion of all papers scoring under 100, resulted in the exclusion of 746 papers. The abstracts of the remaining 109 papers were evaluated and processed, and led to the exclusion 78 papers that were considered to be not relevant to this study. In the last step, the full texts of 31 papers were analyzed, in which 9 were discarded, 2 of them for being paid-for, for which reading was prevented. Thus, 22 papers were selected for a detailed review and categorization.

A detailed review was performed in three colored steps in Figure 1 as follows: general review, review of abstracts, and review of selected papers. The results of these detailed reviews are presented in the following sections.

## 3. General Review

In this section, the results of the general review of search queries performed are presented. The number of papers referring to vulnerabilities, defenses, attacks, or the intersection of these three main topics is presented.

**Figure 2:**
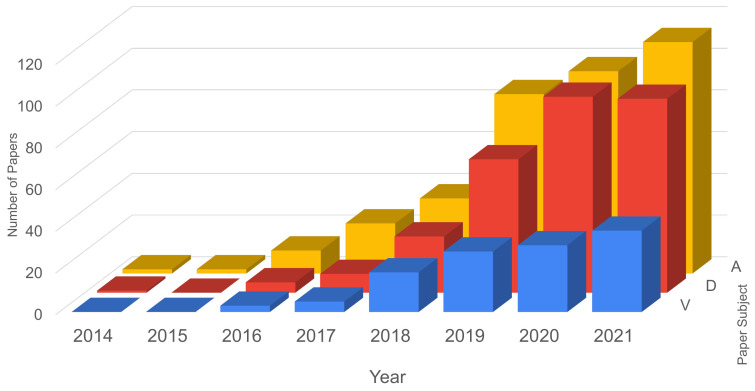
General Review—number of papers focusing on vulnerabilities, attacks and defenses.

Figure 2 presents the number of papers for each year, with respect to security vulnerabilities, attacks and defenses strategies. In 2021, the current year, the number of papers was projected based on the temporal behavior of the types of papers. From the numbers obtained, it can be verified that:

- from 2014 to 2015 the number of papers regarding vulnerabilities where none;
- from 2014 to 2015 only one paper was published regarding defense strategies or mechanisms;
- the number of papers regarding attacks was constant until 2015, when it increased until now;
- papers about defense had a strong increase in number, per year, from all subjects analyzed, but the forecast for 2021 is to maintain, approximately, the previous year’s number;
- the subject of attack presented the greatest number of papers, with 97 papers in 2020, and an estimate of 111 papers for 2021;
- the number of papers addressing vulnerabilities, despite growing over the years, has reduced compared to other subjects;
- the biggest year of growth in papers published was 2019, with a median factor of 2.3 times.

**Figure 3:**
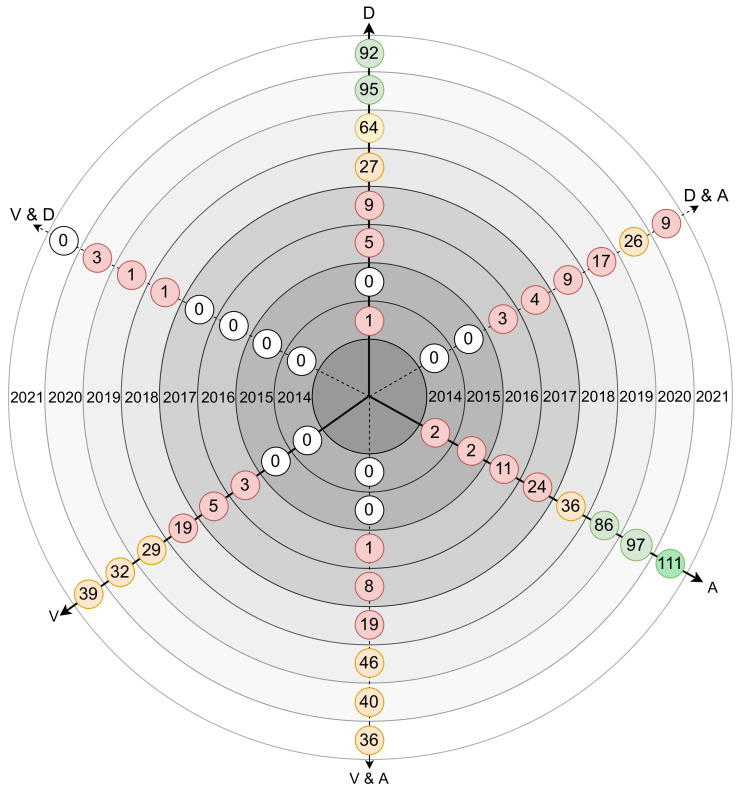
Number of attacks, vulnerabilities and defenses-related papers on multiple axis per year.

Figure 3 displays the number of papers found per year, regarding vulnerabilities and defenses, attacks, and regarding the intersection of these three main topics, Vulnerabilities and Defenses (V&D), Defenses and Attacks (D&A), and Vulnerabilities and Attacks (V&A). From the results obtained, it can be verified that:

- the number of papers addressing attacks is the largest, followed by the number of papers including defense mechanisms.
- the number of papers talking about attacks has been growing significantly
- the number of papers talking about vulnerabilities has grown over the years, despite being small, compared to attacks and defenses
- the number of papers for both topics is twenty five (35)
  from 2014 to 2016.
- between 2017 and 2021 it is possible to identify that the number of papers increase, for all topics, except for the topic of V&D, in which there is a small increase.

**Figure 4:**
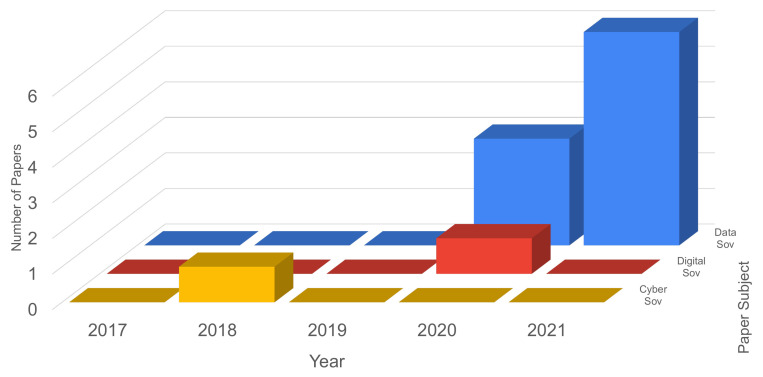
Data sovereignty, digital sovereignty and cyber sovereignty papers results.

Since the queries were extended to included recent areas and possible trends, the results of search queries including data, digital and cyber sovereignty were processed and analyzed. These results are presented in Figure 4. From these results, it can be verified that:

- from 2014 to 2017 for the categories of data, digital and cyber sovereignty, no papers were found.
- in 2018, the number of papers found was only 1 for the category of cyber sovereignty, which did not have papers found again until 2021 (projection).
- in 2019, no papers were found for the three categories.
- for digital sovereignty papers were found only in 2020.
- for data sovereignty, from 2014 to 2019 the number of papers is zero, but an increase is seen in 2020 and 2021 (projection).

From the 11 papers referring to data, digital or cyber sovereignty, two of them focus on particular initiatives, namely the GAIA-X and the ids. In [20] the authors claim that a war for industrial data is starting, Europe is the main battleground, and future platforms need to be built to harness data as close as possible to its production location. To this end, they refer that the GAIA-X, a project initiative launched by the European Union to develop a data infrastructure and data-related service providers for Europe, which intends to tackle this challenge by meeting the highest standards in terms of digital sovereignty and aiming to foster innovation. Data and services are envisioned to be available, grouped and shared in a trusted environment. This paper also refers to the ids initiative, created by the idsa, which consists of a global reference architecture standard, to create and operate virtual data spaces. This architecture is based on commonly recognized data governance models that facilitate the secure exchange and easy linkage of data within business ecosystems. ids pretends to respond to GAIA-X challenges and, in this article, the functioning of its architecture and interconnections are explained. In Ref. [21], the authors also focus their research work around ids described as a virtual data space that uses common standards and governance models to facilitate the secure exchange and easy linkage of data across business ecosystems. It provides a foundation for the creation and use of intelligent services and innovative business processes, while ensuring the digital sovereignty of data owners.

Given all the results regarding the General Review, the research questions formulated can be answered as follows:

- RQ1—What is the progress of the cybersecurity area for Industry 4.0, in number of papers, relative to vulnerabilities, defense and attacks topics? Answer — The results obtained show that the number of papers for the three topics (Vulnerabilities, Attacks and Defenses) have increase from 2014 to 2020. In the current year of 2021, the projection is that the number will be greater for Attacks and Vulnerabilities topics, and the projection for the Defense topic is to be similar to the last year.
- RQ2—What is the progress, in number of papers for the intersections of the topics of vulnerabilities, attacks and defense mechanisms for Industry 4.0? Answer — Regarding the intersection topics, a strong increase of papers was verified regarding V&A, followed by D&A, and, with a slight increase, the V&D.
- RQ3—What is the progress of the number of papers for challenges and trends related to data sovereignty, digital sovereignty and cyber sovereignty areas for Industry 4.0? Answer — With the results obtained, it is concluded that digital, cyber and data sovereignty are relatively recent topics that presumably will grow over the years, particularly data sovereignty. Additionally, GAIA-X and ids are initiatives taking shape and intend to tackle the data sovereignty-related challenges.

## 4. Review of Abstracts

In this section, a review of the abstracts is presented. The papers with a score greater than or equal to 100 were selected and examined.

Figure 5 presents the number of papers per year, out of 109, regarding security vulnerabilities, attacks and defense keywords.

Figure 6 shows the number of papers found per year, with a score equal to or greater than 100, on vulnerabilities and defenses, attacks and on the intersection of these three main topics, Vulnerabilities and Defenses (V&D), Defenses and Attacks (D&A) and Vulnerabilities and Attacks (V&A). From the results, it can be verified that:

- the number of papers referring to defense mechanisms has grown in recent years, and reaches 24 for 2021 (forecast).
- there are no papers matching V&D in any of the years in review.
- between 2014 and 2016, no papers were found.
- between 2017 and 2021, the number of papers increases for all topics, with the exception of V&D, which remains null.

**Figure 6:**
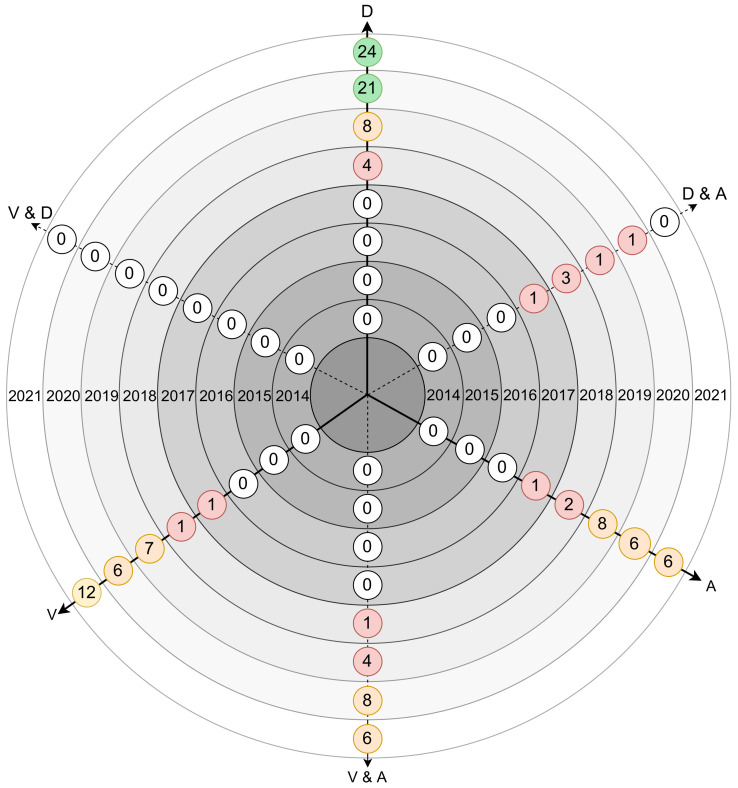
Number of attacks, vulnerabilities and defenses-related papers on multiple axis per year.

In Table 3 papers are categorized according to their score and regarding the occurrence of the Attacks, Defenses, Vulnerabilities keywords. From the results, it can be verified that:

- there are no papers that address vulnerabilities and defenses simultaneously
- the largest group of papers have scores from 100 to 130
- the highest-scoring papers match the three topics: attacks, defenses and vulnerabilities and addresses a risk-assessment of cyber-attacks and defense strategies for Industry 4.0. This paper also reviews the most common cybersecurity vulnerabilities and defense strategies regarding Industry 4.0, in corporate and end-user dimensions.

**Table 3:** Paper Score and Categorization for the Review of Abstracts.

Score	Papers	Attacks	Defenses	Vulnerabilities
100–130	[22,23,24,25,26,27,28,29,30,31,32,33,34,35]			•
	[36,37,38,39,40,41,42,43,44,45,46,47,48,49,50,51,52,53,54,55,56,57]		•	
	[58,59,60,61,62,63,64,65,66,67,68]	•		
	[69,70,71,72,73,74,75,76,77,78]	•		•
	[79,80,81,82,83]	•	•	
	[83,84,85,86,87,88]	•	•	•
131–160	[89,90]			•
	[89,90,91,92,93,94,95,96,97,98]		•	
	[99,100,101,102]	•		
	[69,103,104,105,106,107]	•		•
	[108]	•	•	•
161–190	[109]			•
	[93,110,111,112,113,114,115,115]		•	
	[103,116,117]	•		
	[118,119]	•	•	
	[120]	•	•	•
191–220	[121,122]			•
	[123]	•		
	[124]	•		•
221–250	[125,126,127,128]		•	
251–260	[129]	•	•	•

## 5. Review of Selected Studies

**Figure 7:**
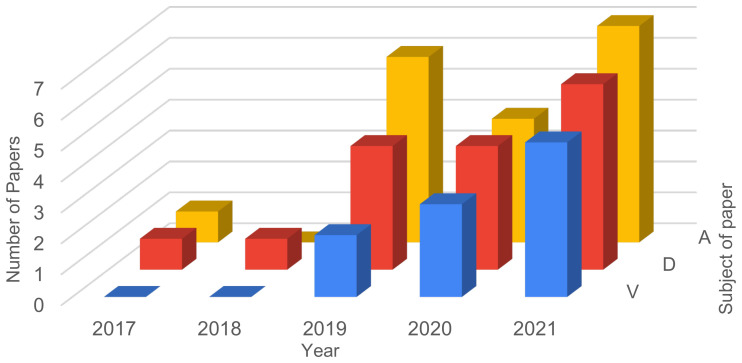
Review of Selected studies—number of papers focusing on vulnerabilities, attacks and defenses.

In this section, a more specific analysis is performed regarding the 22 papers selected after the full text analysis step of the systematic review. In Figure 7 the number of papers for each year regarding the three main topics is presented: vulnerabilities, attacks and defenses. The results for 2021 are projected based on the numbers to date.

A finer analysis regarding the three main topics was performed and, following a similar approach of the previous works in [63,72], a set of categories were defined to fit the results for the selected studies as follows.

### 5.1. Attacks

In this section, 14 relevant papers were found. As shown in Table 4, the attacks studied were grouped in 6 categories: Network, Web Application, System, Devices, Malware, and Social Engineering attacks.

- **Network**
  Network attacks are commonly designed to impact a network’s performance. To impact the performance, a malicious actor can perform a dos [31,63,64,76] attack. This attack consists of generating huge amounts of bogus traffic towards a network with the intent of denying the service to the real users. ddos is a variation of the previous attack, and is the use of a Botnet, also called zombies, which are computers under control of the hacker (e.g., Mirai IoT Botnet [76]) to augment the attack efficiency, using the zombies to generate bigger amounts of traffic directed towards the target, with higher possibilities of rendering it ineffective [62,75,85,103,105,107,129]. Jamming [75] is an attack intended to disrupt network availability. mitm [64,75,85], replay attack [75,108], selective forwarding attack [75], and sybil attack [63,75,85] are all attacks performed on networks, the first, mitm, is the interception of communications by an unintended user. The intercepted traffic can be used to perform other attacks or can be used for gathering sensitive data. Replay attack, also known as playback attack, is an attack in which the traffic gathered by the hacker is repeated maliciously. A Sybil attack consists of multiple fake identities being used to generate additional node identities capable of receiving and forwarding data from and to the victim.
- **Malware**
  Malware is intended to cause damage, steal or modify information in a target computer. This type of attack can be presented in many shapes or forms, such as a virus [103,129], which is a stealth piece of code made to be executed on a victim computer, replicate and propagate into other victims. Worms [63,75,103,129] are also malicious code made to propagate in a network or through emails with the intent of gaining access to the victim computer. Trojan horses [103,129] are another type of malware, designed not to be suspicious when executing, generally disguised as legitimate software. One type of malware that distinguishes itself from the others is ransomware [63,76,107,129]. Ransomware is made to hijack the victim computer, encrypting all the data on the hard drive and requesting a ransom for the decryption of the computer data.
- **Web Application**
  To access sensitive data, web application attacks can lead to compromises in a company’s network. Metadata spoofing and sql Injection are types of attacks that threaten security in iiot. Metadata spoofing is when an attacker modifies a database and causes its integrity to be compromised [76]. When the attackers use sql commands to steal contents within a database, taking advantage of sql injection vulnerabilities, some attacks may occur, such as remote command execution, information disclosure, and authentication bypass [76].
- **System**
  Attacks targeting the ics have increased by 110% since 2016 [75]. For Industry 4.0 systems, the Stuxnet virus [63,75] was detected as a more visible security incident that exploited vulnerabilities in scada [31,72]. Other attacks such as false logic, zero-day and deception attack can target the ics. False logic attack is one attack that could affect scada systems to disrupt control [75]. Zero-day attacks are when a vulnerability is discovered by a hacker and not publicly disclosed, and this type of attack can sabotage scada and power transmission systems [75]. Deception attacks affecting scada and dcs are where the hacker makes the worker accept as true an incorrect scenario to degrade system performance [75].
- **Devices**
  Devices for Industry 4.0, such as sensors, robots and industrial machines, can be attacked by various types of attacks. Physics, measurement injection, side channel and time delay are the most common attacks. Physical attack is when an untrusted worker gains physical access and makes unwanted modifications to devices. Measurement injection is when false data are injected into the sensors. In a side-channel attack, the attacker can gather sensitive information from the device by measuring side-channel information. A time delay attack can disturb the stability of all the industrial control system, by adding extra time delays into measurements and control commands [75].
- **Social Engineering**
  An attacker using social engineering often uses his abilities to convince the user [72,129]. In Ref. [107], the German Steel Mill Cyberattack is detailed. The attackers used a spear-phishing attack which consisted of sending a targeted email from an apparently trusted source to prompt the target to open an illicit attachment or visit a malicious website, where malware is downloaded to their computer to access the corporate network. Another type of social engineering is the phishing attack, different from spear-phishing attack by not having a specific target but a group of targets. The phishing method spoofs the sites of publicly known and trusted organizations and institutions, and allows users to log into these fake websites [129], stealing their credentials.

### 5.2. Vulnerabilities

Regarding vulnerabilities, seven (7) relevant papers were found. The vulnerabilities studied were divided into 4 categories Web Application, Devices, Network, and Authentication, as presented in Table 5.

- **Web Application**
  Vulnerabilities related to web services and applications are usually associated with coding errors that enable destructive or non-destructive attacks. Vulnerabilities that allow malicious users to execute unwanted scripts [25], such as Insecure Deserialization, xxe, csrf, xss and sql Injection in a web application are the most common. Insecure Deserialization occurs when user-controllable data are deserialized by a website, allowing attackers to manipulate serialized objects to pass harmful data to the application code. xxe is a vulnerability that allows an attacker to interfere with an application’s XML data processing, enabling him to view files on the application server’s file system and interact with any back-end or external systems that the application itself can access. csrf is a web security vulnerability that allows an attacker to trick users into taking actions they do not intend to take. xss is a vulnerability that allows an attacker to compromise the interactions that users have with a vulnerable application, in which malicious scripts are injected into sites that are not marked as trusted. sql Injection is a vulnerability that allows an attacker to interfere with the queries an application makes to your database.
- **Devices**
  With regards to iiot devices, such as plc, cameras, smart-routers, smart-meters from various vendors, vulnerabilities were found in the software and firmware of the devices. These are Buffer Overflow, Infinite Loop, uaf, Heap Overflow and ddos vulnerabilities [105]. Buffer overflow happens when a program or process attempts to write more data to a fixed-length block of memory, or buffer, than the buffer is designed to hold. Infinite Loop happens when an iteration or loop implemented in the program cannot reach the exit condition. If an attacker can manipulate the loop, it could allow him to force the device to consume excessive resources, e.g., CPU and RAM. uaf vulnerability is related to incorrect use of the dynamic memory, the program does not clear the pointer to the freed memory location, enabling the attacker to cause an error on the program. Heap Overflow is a type of buffer overflow vulnerability, but this one happens on the heap data area. Vulnerabilities such as password leakage and password hash cracking are also vulnerabilities that attack iiot devices [76]. Backdoors are also found on these devices, in which they are classified as a special vulnerable point in software or firmware analysis, which is different from traditional vulnerabilities or bugs in control-flow types [105].
- **Network**
  Network vulnerabilities are directly related to attacks to devices and services [72]. According to the authors in [71], Industry 4.0 has created new scenarios of cyber-threats designed for classic it. To cope with this problem the authors address the security issues related to covert channels applied to industrial networks, identifying vulnerability points when classic it converges with operational technologies such as edge computing infrastructures. The authors define the strategy of attack starting by exploiting the tcp protocol to set up a covert channel and then proceeding to more active and offensive methods to exploit a real industrial iot test bed.
- **Authentication**
  According to [25] improper authentication allows malicious users to access sensitive data through dictionary, birthday and brute force attacks focused on in [75,76]. Dictionary attacks are only possible due to the improper setup of authentication, allowing attackers to perform countless login tries with every word from a word list. Birthday attack comes from the birthday paradox, where it was used to create password hash collision. Brute force is an attack of trial and error, where attacker use every available resource to guess login credentials and encryption keys, among others.

### 5.3. Defenses

Regarding defense mechanisms, 8 relevant papers were found. The defense mechanisms studied were divided into the following 6 categories, Application, Devices, Network, Social Engineering, Policy, and System, as shown in Table 6.

- **Application**—Application defense can be seen as developing custom defense mechanisms to improve overall safety of a product. Various methods can be selected to achieve a certain desired result. The authors in [83] propose a defense mechanism against timing-based side-channel attacks to response time on the Internet of Things. This mechanism follows two modules, vulnerability testing and privacy protection. The result of this proposal, in an experimental scenario, shows that the mechanism created is capable of precisely identify the side-channel leakages related to response time and efficiently mask them. In [119] the authors propose a mhmm for designing threat intelligence that is capable of monitoring and recognizing cyber-attacks for Industry 4.0 systems. The mechanism developed showed the capability of completely discovering physical and network attacks using physical power systems and UNSW-NB15 datasets [131]. In terms of performance, the mechanism outnumbered five different peer techniques in terms of detection rates, false positives and processing times. This paper presents a useful mechanism to be deployed in any Industry 4.0 scenario to assess cybersecurity threats. In study [38], a framework is proposed, which consists of a Blockchain-based model distributed through an SDN-IoT-enabled architecture to ensure adequate security, which is the main concern in Industry 4.0. With Blockchain integration, the authors ensure that all data are safe for Industry 4.0 applications.
- **Devices**—All devices and software used in organizations should be configured safely, and controlled changes should be ensured [62]. In Ref. [105], the authors present a framework for analyzing and discovering vulnerabilities in iot and Industry 4.0, called *VulHunter*, which aims to discover unknown vulnerabilities in the analysis based on the patch of known vulnerabilities. To secure network devices, the authors in [62] address systems that perform identity checks and data packets, which can be used against attacks on routing tables. In Ref. [86] the authors present the framework IIoT-SIDefender, to measure security of sensitive information leakage and leverage in every layer of iiot devices. The results of the framework demonstrate that the leakage points of sensitive information can be detected, and attacks can also be defended with real-time hot fixes generated to prevent such attacks. In Ref. [130] ai is seen as a means of threat detection in devices and the combination of attack surface reduction, secure development life cycle, data protection, secure and hardened device hardware and firmware, and machine learning may be critical in moving forward with a secure, vigilant and resilient Industry 4.0-enabled devices.
- **Network**—Traffic between networks with different security levels should be restricted and monitored [62]. With the aim of ensuring communications between industry 4.0 supply chain partners, the authors in [64] propose an efficient tls-based authentication mechanism that is resistant to mitm attacks for web applications that use the tls protocol to protect HTTP communications. The proposed mechanism prevents the attacker from impersonating the legitimate server for the user to guarantee confidentiality. The authors in [87] aim to identify and map potential vulnerable endpoints in an industrial paradigm and propose a robust way of securing a wireless sensor network, ensuring the integrity and authenticity of data acquisitions. During the case study, the authors identify technologies used in the Industry 4.0, and their respective security mechanisms, possible attacks, solutions and vulnerable points, one being on daq devices, and on the Communication Layer between daq and the cloud. In Ref. [108], the authors propose a defense framework based on sdn that consists of a model for traffic management and anomaly detection. Based on this framework, the authors studied the use of this methodology for iot networks and for scada systems [84], in which it allowed the extraction of traffic patterns to detect and prevent various network attacks such as arp spoofing, replay attacks and detect malicious command forwarding behaviors.
- **Social Engineering**—Defense strategies for social engineering can include collaborators training to recognize sensitive information requests (phone numbers, emails, etc.) or a redirection to false web sites that intend to capture this data outside the scope of the corporation. For the authors in [129], the use of *Truecaller* or *Dialer* Software is seen as a method of defense against phishing attacks. According to [62], emails, attachments, and links that appear suspicious should be used with caution or avoided; these links should be double-checked and/or typed directly into the browser, to reduce the risk of attack.
- **Policy**—In Ref. [129], authors state that institutions that intend to implement Industry 4.0 architectures must determine first the information security policies, taking advantage of the ISO/IEC 27001 [132] and ISO/IEC 27002 [133] standards. Privacy, integrity and accessibility topics, such as access controls, backups, use of cryptographic controls, human resources security and software installation restrictions should also be included, and inventory of hardware and software assets and security vulnerability management [62,129] must also be taken into account.
- **System**—According to [63], Blockchain technology that uses hash and cryptography algorithms can also be used as a solution against various attacks on iiot systems, such as injection attacks and malware attacks, guaranteeing confidentiality and integrity for databases and Blockchain. In Ref. [103], the authors also use Blockchain, taking into account that it is decentralized and resistant to cyber hacks and can also be incorporated with the smart contract system to increase operational security in the battery’s energy storage systems against cyber-attacks.

## 6. Discussion and Future Challenges

As a result of the systematic review, it is possible to identify the most common types of attacks, vulnerabilities and defense mechanisms for Industry 4.0, and check their progress in recent years. The main highlights that can be drawn from the results are that:

- Regarding attacks: the number of papers was constant until 2015, and has been growing significantly since. Papers addressing attacks are the highest in number, followed by the number of papers including defense mechanisms;
- Regarding defenses: the number of papers had the highest increase in number, per year, from all subjects analyzed, but the forecast for 2021 is to maintain, approximately, the previous year’s number.
- Regarding vulnerabilities: the number of papers addressing vulnerabilities, despite growing over the years, has reduced compared to attack and defenses topics.
- Regarding the correlation between topics (attacks, defenses and vulnerabilities), between 2017 and 2021 it is possible to identify that for all topics, the number of papers had a strong increase, except for the topic of V&D, which had a small increase.

Regarding selected studies on attacks categorized in Table 4, it can be concluded that the largest number of attacks for Industry 4.0 can occur as network attacks, in which it can be identified that dos, ddos and mitm are the most common attacks to be taken into account. It is also identified that attacks have been increasing over the years since 2017.

Regarding selected studies on vulnerabilities categorized in Table 5, it is possible to conclude that with the advancement of technology, the number of threats has increased. The most relevant vulnerabilities were identified in the *Device* categories and in the *Networks* category. Buffer overflows, ddos vulnerabilities and backdoors are the most common vulnerabilities found in devices, and network vulnerabilities are directly related to attacks on devices and services.

Regarding selected studies on defenses categorized in Table 6, it can be concluded that defense mechanisms tend to evolve in the face of attacks that respond to them. Blockchain was one of the most relevant technologies studied, to guarantee the security of some attacks. Cryptography mechanisms were also addressed in this study as being an effective method, e.g., for mitm attacks.

From the results obtained regarding data sovereignty, digital sovereignty and cyber sovereignty, it can be concluded that these areas are very recent. The terms “digital sovereignty” and “cyber sovereignty” were less recurrent and, on the other hand, the term “data sovereignty” seems to be a trend for cybersecurity in the context of Industry 4.0. These concepts, focused on secure sharing and owning data, are being addressed and the research work in this area seems to be growing (by the progress of the published papers), while recent initiatives such as ids and GAIA-X are also being promoted. Thus, the following set of future challenges for corporations and industries can be depicted:

- Enable intra-exchange and inter-exchange of industrial data, taking into account the full value of exchanged data;
- Enable secure data exchange within particular corporation scopes and time frames, while maintaining the control on the data providers;
- Exercise control of data over devices and the Internet, taking into account industrial spaces and borders;
- Massive data sharing within sectors or groups of companies;
- Allow secure data ownership transfer between sectors or groups of companies;
- Perform real-time analysis of massively generated data;
- Enforce personal data privacy mechanisms when sharing data between companies;
- Enable sharing of secure and trusted data based on standards;
- Share information in an environment of trust between producers and consumers;
- Allow interoperability for business ecosystems;
- Enable data sharing between companies to enhance corporation sustainability;
- Enforce self-determined control of data use (data sovereignty) while offering new ”smart services” and innovative business processes across companies and industries.

## 7. Conclusions

Security incidents may impact industries that are progressing and deploying Industry 4.0 concepts and, since recent technologies such as ai or iot are being included in Industry 4.0, the progress of these implementations requires constant surveillance regarding cybersecurity. At the same time, it is also important to forecast cybersecurity-related challenges and trends to better build and adapt these implementations to possible future attacks and their impacts.

This paper reviews recent research efforts on attacks, vulnerabilities, and defenses in Industry 4.0 implementations, and highlights security-related topics and challenges that seem to be surging in this area. The contributions of this paper can be divided in the following: (1) it presents a systematic survey based on a general and abstract analysis, (2) it analyzes in detail and categorizes selected studies on attacks, vulnerabilities and defenses mechanisms, and (3) it provides a discussion on recent challenges and trends regarding these areas on Industry 4.0.

From the systematic review results we concluded that attacks, defenses and vulnerabilities in Industry 4.0 implementations are increasing and capturing researcher attention. When categorizing selected studies, we concluded that the greatest number of attacks are network-based attacks, such as dos and mitm attacks, exploiting vulnerabilities in Industry 4.0 networks and devices. Additionally, it is highlighted that topics related to digital sovereignty and mainly data sovereignty appear to be a trend in this area, and ids and GAIA-X are recent initiatives intended to face the challenges related to the data sovereignty topic.

## Figures and Tables

**Figure 5:**
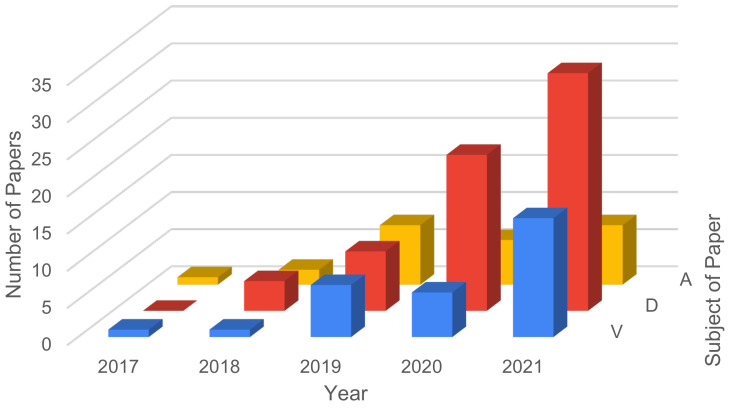
Review of Abstracts—number of papers focusing on vulnerabilities, attacks and defenses.

**Table 1:** Defined Keywords.

Primary Keywords	Secondary Keywords
Industry 4.0	Attack
Industry 5.0	Vulnerabilities
	Defense
	Data Sovereignty
	Digital Sovereignty
	Cyber Sovereignty

**Table 2:** Exclusion criteria.

(1) Papers that are not in English
(2) Papers with a score of less than 100
(3) Papers considered not relevant for this study
(4) Papers that required a special license to access their content

**Table 4:** Attacks.

Attack Type	Description	Paper
Network	An attack intended to access or map a network to cripple its performance or obtain sensitive information.	[31,62,64,76,103,105] [63,75,85,107,129]
Malware	Malicious software created to attack and exploit systems.	[63,76,85,103,107,108,129]
Web Application	Attacks on web services and applications in order to access sensitive data.	[25,76,85]
System	Attacks on control systems and other manufacturing control related devices.	[31,63,72,75]
Devices	Attacks exploiting software and/or firmware of IoT devices.	[25,63,75]
Social Engineering	Physiological manipulation of a victim with the intent of obtaining sensitive information.	[31,107,129]

**Table 5:** Vulnerabilities.

Vulnerability Type	Description	Paper
Web Application	Flaws in web services and applications that can compromise sensitive data and service availability	[25,85,104,105]
Devices	Flaws in source code, capable of allowing unwanted access	[62,85,105]
Network	Flaws capable of enabling network attacks or leaks of sensitive information.	[63,75]
Authentication	Incorrect validation of user credentials	[25]

**Table 6:** Defenses.

Defense	Description	Paper
Application	Mechanisms that enable secure processing in applications.	[38,62,63,64,103,119]
Devices	Mechanisms that enable device security.	[62,86,105,130]
Network	Strategies or software designed to maintain network security and efficiency	[62,64,108]
Social Engineering	Promote training and awareness	[62,129]
Policy	Established rules used in a procedure, protocol or standard	[31,63]
System	Technologies used in order to ensure security in systems	[63,103]
